# Childhood Maltreatment and Longitudinal Epigenetic Aging: NIMHD Social Epigenomics Program

**DOI:** 10.1001/jamanetworkopen.2024.21877

**Published:** 2024-07-29

**Authors:** Olivia D. Chang, Helen C. S. Meier, Kathryn Maguire-Jack, Pamela Davis-Kean, Colter Mitchell

**Affiliations:** 1 School of Social Work, University of Michigan, Ann Arbor; 2 Department of Psychology, University of Michigan, Ann Arbor; 3 Survey Research Center, University of Michigan, Ann Arbor; 4 Population Studies Center, University of Michigan, Ann Arbor; 5 Department of Sociology, University of Michigan, Ann Arbor

## Abstract

**Importance:**

Child physical and emotional abuse and neglect may affect epigenetic signatures of accelerated aging several years after the exposure.

**Objective:**

To examine the longitudinal outcomes of early-childhood and midchildhood exposures to maltreatment on later childhood and adolescent profiles of epigenetic accelerated aging.

**Design, Setting, and Participants:**

This cohort study used data from the Future of Families and Child Wellbeing Study (enrolled 1998-2000), a US birth cohort study with available DNA methylation (DNAm) data at ages 9 and 15 years (assayed between 2017 and 2020) and phenotypic data at birth (wave 1), and ages 3 (wave 3), 5 (wave 4), 9 (wave 5), and 15 (wave 6) years. Data were analyzed between June 18 and December 10, 2023.

**Exposures:**

Emotional aggression, physical assault, emotional neglect, and physical neglect via the Parent-Child Conflict Tactics Scale at ages 3 and 5 years.

**Main Outcomes and Measures:**

Epigenetic accelerated aging (DNAmAA) was measured using 3 machine learning–derived surrogates of aging (GrimAge, PhenoAge, and DunedinPACE) and 2 machine learning–derived surrogates of age (Horvath and PedBE), residualized for age in months.

**Results:**

A total of 1971 children (992 [50.3%] male) representative of births in large US cities between 1998 and 2000 were included. Physical assault at age 3 years was positively associated with DNAmAA for PhenoAge (β = 0.073; 95% CI, 0.019-0.127), and emotional aggression at age 3 years was negatively associated with PhenoAge DNAmAA (β = −0.107; 95% CI, −0.162 to −0.052). Emotional neglect at age 5 years was positively associated with PhenoAge DNAmAA (β = 0.051; 95% CI, 0.006-0.097). Cumulative exposure to physical assault between ages 3 and 5 years was positively associated with PhenoAge DNAmAA (β = 0.063; 95% CI, 0.003-0.123); emotional aggression was negatively associated with PhenoAge DNAmAA (β = −0.104; 95% CI, −0.165 to −0.043). The association of these measures with age 15 years PhenoAge DNAmAA was almost fully mediated by age 9 years PhenoAge DNAm age acceleration. Similar patterns were found for GrimAge, DunedinPACE, and PhenoAge, but only those for PhenoAge remained after adjustments for multiple comparisons.

**Conclusions and Relevance:**

In this cohort study, altered patterns of DNAmAA were sensitive to the type and timing of child maltreatment exposure and appeared to be associated with more proximate biological embedding of stress.

## Key Points

**Question:**

Are longitudinal, developmental influences of child maltreatment during early childhood and midchildhood associated with epigenetic signatures of accelerated aging in later childhood and adolescence?

**Findings:**

In a cohort study of 1971 children across the US, physical assault, emotional aggression, physical neglect at age 3 years, and emotional neglect at age 5 years were associated with epigenetic signatures of accelerated aging. Maltreatment subtypes were differentially associated with patterns of epigenetic age acceleration.

**Meaning:**

The findings of this study suggest that DNA methylation may be sensitive to the type and timing of maltreatment many years after the exposure.

## Teaser

This cohort study examines whether childhood exposure to physical and emotional abuse and neglect is associated with the rate of epigenetic aging.

## Introduction

Approximately 1 of every 7 US children experiences abuse or neglect each year.^1^ Maltreatment poses severe threats to children’s physical, psychological, and socioemotional development^2^ both in childhood and later in life.^3,4,5,6^ The effects of maltreatment are not merely attributable to greater exposure to additional risks surrounding maltreating environments^7^; maltreatment in early life may become biologically embedded, conferring a specific downstream risk for adverse health outcomes across the lifespan.^8,9^

DNA methylation (DNAm) is one potential mechanism by which children’s exposure to adverse experiences may become biologically embedded. As an epigenetic modification to DNA affecting gene expression, DNAm can be influenced by behavior and environment.^9,10^ Three systematic reviews examining childhood maltreatment and DNAm reported mixed support for an association,^11,12,13^ but most extant evidence relies on candidate gene studies,^14,15,16,17,18,19,20,21,22^ which have known replicability issues.^9,23,24,25^

Beyond single loci analyses, the use of epigenetic predictive surrogates to examine alterations in DNAm represents one promising approach to better understand how maltreatment is associated with DNAm.^26^ Surrogates are typically machine learning–derived algorithms from thousands of DNAm measures predicting an exposure, disease, or process.^9^ One prominent set of surrogates, epigenetic clocks, are DNAm signatures predictive of chronological age or aging phenotypes.^27,28,29,30^ Epigenetic age acceleration (DNAmAA) or the age-residualized epigenetic clock is thought to indicate premature (positive residuals) or delayed (negative residuals) biological aging. Epigenetic clocks are trained on different age-related outcomes in different populations.^29^ First-generation epigenetic clocks, including Horvath,^28^ are trained on chronological age, while later clocks, including PhenoAge,^29^ GrimAge,^30^ and DunedinPACE,^31^ are trained on aging-related phenotypes associated with age-related health outcomes, including mortality, chronic diseases, and cognitive decline.^29,30,31,32,33,34,35^

With respect to child maltreatment, limited studies have small sample sizes and cross-sectional associations^36,37,38,39^ or larger cohorts that rely on retrospective reports of maltreatment.^36,40,41,42^ None of these studies could examine the outcomes of changes in methylation, as there are few studies with longitudinal methylation assessments.

In addition to examining the prospective association between different forms of maltreatment and epigenetic age in childhood, a key gap in knowledge is whether maltreatment during periods of developmental sensitivity or the accumulation of maltreatment over childhood is associated with DNAmAA. In line with developmental sensitivity hypotheses,^12,43^ maltreatment occurring earlier in life may be more strongly associated with DNAmAA.^44^ In addition, it is critical to examine how a model of cumulative exposure may influence negative outcomes.^43^ Furthermore, given that child maltreatment represents a distinct adverse childhood event (ACE),^45^ it is necessary to understand its specific association with epigenetic age independent of other ACEs.

## Methods

### Sample

Data were obtained from the Future of Families and Child Wellbeing Study (N = 4898), a birth cohort study representative of births from 20 large US cities in 15 states between 1998 and 2000 in which births to unmarried mothers were oversampled.^46^ A random subset of children had DNAm assays completed at ages 9 and 15 years.^47,48^ Approval for this study was obtained from the University of Michigan Institutional Review Board; informed consent was waived because data were deidentified and had been previously collected. We followed the Strengthening the Reporting of Observational Studies in Epidemiology (STROBE) reporting guideline.

### Measures

#### Child Maltreatment

The Parent-Child Conflict Tactics Scale^49^ reported at ages 3 and 5 years was used to assess 4 types of maltreatment: physical assault (PA) (4 items), emotional aggression (EA) (5 items), physical neglect (PN) (4 items), and emotional neglect (EN) (1 item). Parents responded using a 7-point Likert scale for each statement. Summed scores were constructed using midpoints of the response options: never or not in the past year (0), once (1), twice (2), 3 to 5 times (4), 6 to 10 times (8), 11 to 20 times (15), and more than 20 times (25).^49^ Responses with 2 or more unanswered items were coded as missing. For cumulative exposure analyses, subscales were summed from both ages. Cumulative exposure was coded as missing if either age 3 or 5 years had missing responses.

#### Epigenetic Age Acceleration

Saliva samples were collected at ages 9 and 15 years (Oragene DNA Self-Collection kits; DNA Genotek), and available samples (n = 2020) were assayed using methylation arrays (Infinium Human Methylation 450K [n = 828] and EPIC [n = 1143], Illumina) (eMethods in Supplement 1).^47,48^ Samples were excluded if the ENmix R package quality control procedure identified outlier methylation or bisulfite conversion values or if the DNAm-predicted sex differed from the survey-recorded sex, resulting in a final analytic sample of 1971 youth at age 9 years and 1906 youth at age 15 years. Demographic comparisons of the larger Future of Families and Child Wellbeing Study sample and the DNA assay subsample are provided in eTable 1 in Supplement 1.

Analyses were focused primarily on 3 epigenetic clocks trained on aging: PhenoAge,^29^ GrimAge,^30^ and DunedinPACE.^31^ Because those clocks were trained in older adult blood samples, we also included 2 chronological age–trained clocks: Horvath^28^—a cross-tissue predictor of age—and PedBE^50^—a buccal tissue–trained predictor of chronological age in children. The DNAmAA measure was generated by regressing chronological age on each clock and standardizing the residual, denoted with age acceleration appended to each clock.

#### Covariates

Covariates included child’s assigned sex at birth, city of birth, maternal educational attainment at baseline, maternal age at birth, maternal marital status at birth, poverty-to-income ratio at birth, prenatal smoking, child internalizing and externalizing problems measured via the Child Behavior Checklist 2-3,^51^ child chronological age in months, assay type (450K or Illumina EPIC), cell type proportions of the sample estimated using EpiDISH,^52^ and self-reported race and ethnicity (any race Hispanic, non-Hispanic Black, multiracial [investigator observed when parents reported >1 race and ethnicity], non-Hispanic White, and Other. Other (American Indian, Asian, and Pacific Islander) is reported by the Future of Families and Child Wellbeing Study due to small sample size. Race and ethnicity were included as covariates given their association with the exposures (childhood adversity). Other ACEs, not including child maltreatment, were assessed by the mother’s report of parental mental illness (Composite International Diagnostic Interview-Short Form)^53^, parental substance use, mother’s exposure to intimate partner violence (revised Conflict Tactics Scale^49^), parental criminal justice system involvement, and parental death at ages 3 and 5 years. Summed scores were constructed at age 3 years such that higher scores indicated greater exposure to ACEs. Summed scores were similarly constructed at age 5 years, with the exception that parental anxiety and substance use were not assessed at that time, and thus were not included. For cumulative exposure analyses, ACEs were summed across both ages.

### Statistical Analysis

Data analysis was performed from June 18 to December 10, 2023. We first examined covariance matrices of the parenting measures and the epigenetic clocks, correlated using the Pearson correlation coefficient independently. Next, multiple linear regression using full-information maximum likelihood estimation to address missingness on independent variables was conducted to estimate the association of maltreatment with each epigenetic clock. Model 1 adjusted for age, assay type, cell proportions, internalizing and externalizing problems, sex, city, race and ethnicity, educational level, maternal age, maternal marital status, prenatal smoking, and poverty-to-income ratio. Model 2 additionally adjusted for other ACEs. Each model was tested separately using scores at ages 3 and 5 years, as well as cumulative scores across ages 3 and 5 years. Model 0 with only age and epigenetic controls provides a less conservative estimate of the associations. Subsequently, models of age 3 and 5 years and accumulation of ages 3 and 5 years and their association with DNAmAA at age 15 years with and without age 9 years DNAmAA acceleration data provides a test of the continuing influence of early-childhood and midchildhood maltreatment. As a sensitivity analysis, we also tested an omnibus accumulation model wherein we summed across all types of maltreatment reported between ages 3 and 5 years. All analyses were conducted in Stata, version 17.0 (StataCorp LLC), and we used 2-sided *t* tests in multiple linear regression with a Benjamini-Hochberg procedure to account for multiple testing. The Benjamini-Hochberg procedure was applied to adjust *P* values obtained from the analysis of associations between 4 different child maltreatment types from 3 different exposure periods with DNAm accelerated aging on 3 clocks (GrimAge, DunedinPACE, and PhenoAge) at year 9. In this study, we chose to use a false discovery rate threshold of 0.30, which allowed us to capture more potential associations for further investigation while still controlling the rate of false discoveries.

## Results

A total of 1971 children (992 [50.3%] male, 979 [49.7%] female, 889 [45.1%] Black, 425 [21.6%] Hispanic, 281 [14.3%] multiracial, 344 [17.5%] White, and 32 [1.6%] Other) were included in the full analytic sample. Sample descriptives and distributions of study variables, including missingness due to nonresponse, are presented in Table 1.

**Table 1.** Descriptive Statistics of Study Variables

Variable	Full DNA assay sample (N = 1971)	
	No. with data (% missing)	Mean (SD) or No. (%)^a^
Adverse exposures		
PA at age 3 y	1585 (19.6)	9.31 (12.51)
PA at age 5 y	1535 (22.1)	7.24 (11.04)
EA at age 3 y	1586 (19.5)	25.58 (20.29)
EA at age 5 y	1536 (22.1)	26.64 (20.51)
PN at age 3 y	1586 (19.5)	0.28 (2.94)
PN at age 5 y	1531 (22.3)	0.21 (1.59)
EN at age 3 y	1586 (19.5)	0.32 (1.89)
EN at age 5 y	1531 (22.3)	0.27 (1.61)
Other ACEs at age 3 y^b^	1521 (22.8)	1.00 (0.90)
Other ACEs at age 5 y^b^	1837 (6.8)	1.02 (0.85)
Child characteristics		
Internalizing behavior	1784 (9.5)	0.33 (0.21)
Externalizing behavior	1613 (18.2)	0.52 (0.27)
Child sex, No. %		
Male	1971 (0)	992 (50.3)
Female		979 (49.7)
Race and ethnicity, No. %		
Black, non-Hispanic	1971 (0)	889 (45.1)
Hispanic		425 (21.6)
Multiracial^c^		281 (14.3)
White, non-Hispanic		344 (17.5)
Other^d^		32 (1.6)
Parent and family characteristics		
Mother’s age, y	1970 (0.1)	25.29 (5.98)
Prenatal smoking, No. %	1968 (0.2)	376 (19.1)
Educational level, No. %		
Less than high school	1967 (0.2)	616 (31.3)
High school degree or GED		607 (30.9)
Some college or technical school		520 (26.4)
College degree or higher		224 (11.4)
Mother’s relationship to child’s father, No. %		
Single	1971 (0)	770 (39.1)
Cohabitating		715 (36.3)
Married		486 (24.7)
Poverty-to-income ratio	1971 (0)	2.34 (2.49)

Abbreviations: ACEs, adverse childhood events; EA, emotional aggression; EN, emotional neglect; GED, general educational development; PA, physical assault; PN, physical neglect.

^a^ Mean (SD) for continuous variables; No. (%) for categorical variables.

^b^ Other ACEs reflects the count of ACEs excluding maltreatment.

^c^ Multiracial includes children whose parents reported more than 1 race or ethnicity.

^d^ Other includes American Indian, Asian, and Pacific Islander as reported by the Future of Families and Child Wellbeing Study due to small sample size.

**Figure 1.**
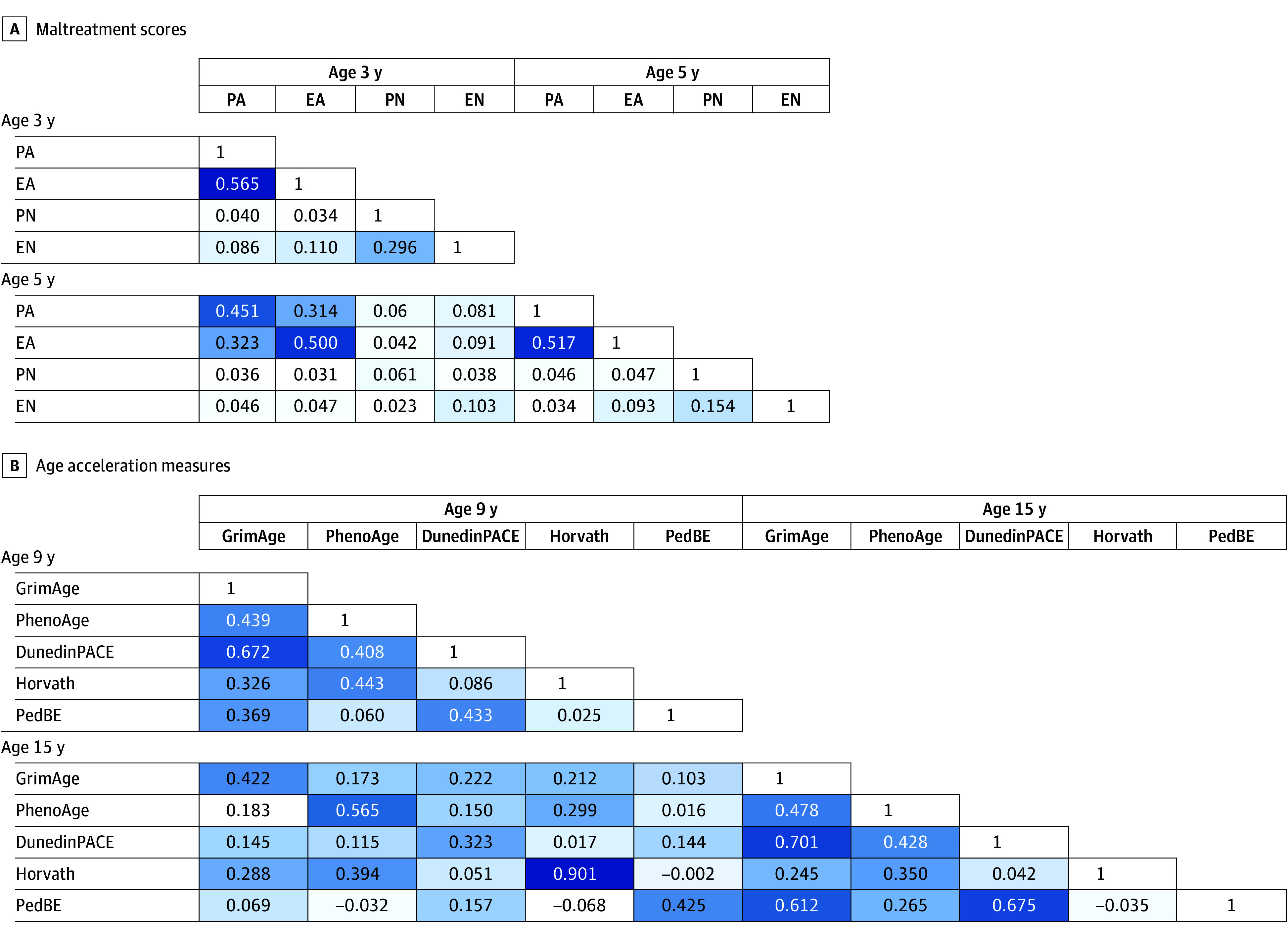
Pearson Correlation Matrix and Heat Plot of Key Measures A, Maltreatment scores at ages 3 and 5 years. B, DNA age acceleration measures at ages 9 and 15 years. Darker color indicates stronger correlation. EA indicates emotional aggression; EN, emotional neglect; PA, physical assault; and PN, physical neglect.

Figure 1 shows the correlation structure of parent-reported child maltreatment and DNAmAA. As shown in Figure 1A, PA and EA were moderately correlated with each other at each age and over time (mean correlation, 0.52). Measures of neglect had low to no correlation with any of the other measures of maltreatment. In contrast, the age 9 and 15 years DNAmAA measures (Figure 1B) were moderately correlated with each other and their repeat measure. The aging phenotype clocks were more correlated with each other than the chronological clocks.

### Child Maltreatment and Epigenetic Aging at Age 9 Years

**Figure 2.**
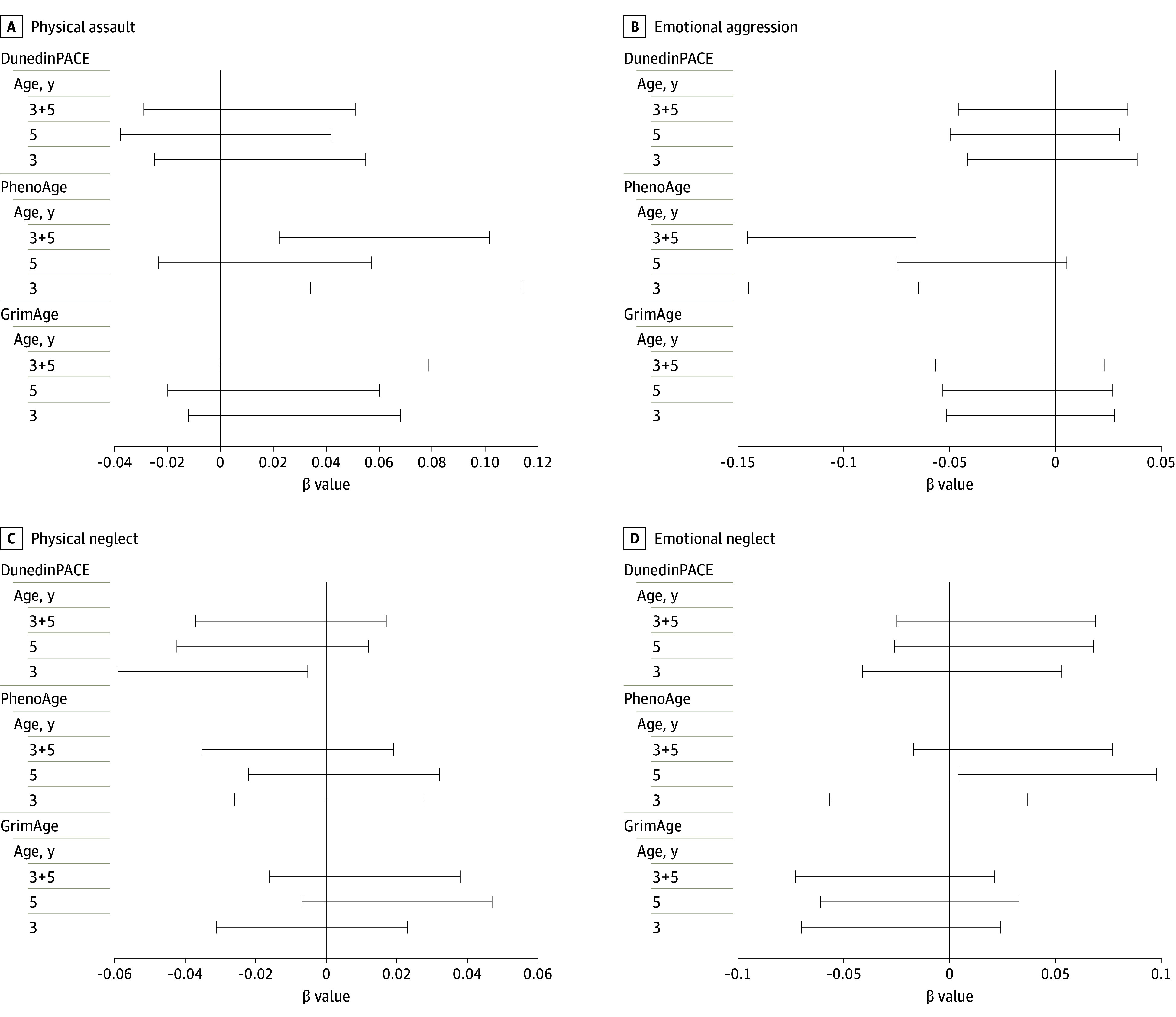
95% CIs of the Association Between Maltreatment Types Findings are shown for physical assault (A), emotional aggression (B), physical neglect (C), and emotional neglect (D) at ages 3 and 5 years, accumulated from ages 3 to 5 years, and age acceleration estimates. Models were adjusted for age, assay type, cell proportions, internalizing and externalizing problems, sex, city, race and ethnicity, educational level, maternal age, maternal marital status, maternal prenatal smoking, and poverty-to-income ratio. Data are presented in Table 2.

The developmental and maltreatment type associations with epigenetic aging at age 9 years are presented in Table 2 and Figure 2. In examining the 3 aging clocks (GrimAge, DunedinPACE, and PhenoAge), we observed a general pattern of PA resulting in higher DNAmAA, but after adjusting for multiple comparisons and age, assay type, cell proportions, internalizing and externalizing problems, sex, city, race and ethnicity, educational level, maternal age, maternal marital status, prenatal smoking, and poverty-to-income ratio, only PA at age 3 years was positively associated with PhenoAge age acceleration (β = 0.073; 95% CI, 0.019-0.127). Emotional aggression at age 3 years was negatively associated with PhenoAge age acceleration at age 9 years (β = −0.107; 95% CI, −0.162 to −0.052). Even after additional adjustment for other ACEs at age 3 years (eTable 2 in Supplement 1), PA at age 3 years was associated with PhenoAge age acceleration (β = 0.074; 95% CI, 0.020-0.128), and EA at age 3 years was associated with PhenoAge age acceleration (β = −0.105; 95% CI, −0.160 to −0.049). In the age 5 years model, PA and EA associations with PhenoAge age acceleration were in the same direction, but the findings were not significant after adjusting for multiple testing and controls.

**Table 2.** Associations Between Child Maltreatment Exposure and DNAmAA Measures at Year 9 (Model 1)^a^

Maltreatment exposure	Year 9 DNAmAA, β (95% CI)				
	Horvath	PedBE	GrimAge	PhenoAge	DunedinPACE
**Year 3**					
PA	0.029 (−0.030 to 0.087)	<−0.001 (−0.048 to 0.048)	0.027 (−0.012 to 0.066)	0.073 (0.019 to 0.127)	0.014 (−0.023 to 0.052)
EA	−0.044 (−0.104 to 0.016)	0.052 (0.002 to 0.101)	−0.015 (−0.055 to 0.026)	−0.107 (−0.162 to −0.052)	−0.004 (−0.042 to 0.034)
PN	−0.053 (−0.104 to −0.002)	0.009 (−0.033 to 0.050)	−0.004 (−0.038 to 0.030)	0.002 (−0.045 to 0.048)	−0.032 (−0.064 to <−0.001)
EN	−0.004 (−0.055 to 0.047)	−0.002 (−0.044 to 0.039)	−0.025 (−0.059 to 0.009)	−0.012 (−0.059 to 0.035)	0.005 (−0.027 to 0.037)
**Year 5**					
PA	0.036 (−0.022 to 0.093)	−0.002 (−0.048 to 0.044)	0.019 (−0.018 to 0.056)	0.018 (−0.034 to 0.070)	0.002 (−0.034 to 0.037)
EA	−0.036 (−0.096 to 0.024)	0.029 (−0.019 to 0.077)	−0.014 (−0.052 to 0.025)	−0.034 (−0.088 to 0.020)	−0.011 (−0.048 to 0.027)
PN	−0.022 (−0.073 to 0.028)	−0.012 (−0.052 to 0.028)	0.020 (−0.012 to 0.053)	0.005 (−0.040 to 0.051)	−0.015 (−0.046 to 0.016)
EN	−0.004 (−0.055 to 0.047)	0.004 (−0.036 to 0.044)	−0.015 (−0.047 to 0.018)	0.051 (0.006 to 0.097)	0.021 (−0.010 to 0.052)
**Years 3 and 5**					
PA	0.048 (−0.017 to 0.114)	−0.002 (−0.055 to 0.050)	0.037 (−0.006 to 0.080)	0.063 (0.003 to 0.123)	0.010 (−0.032 to 0.051)
EA	−0.063 (−0.130 to 0.005)	0.055 (0.001 to 0.109)	−0.021 (−0.065 to 0.023)	−0.104 (−0.165 to −0.043)	−0.008 (−0.051 to 0.035)
PN	−0.026 (−0.081 to 0.028)	0.013 (−0.030 to 0.057)	0.008 (−0.028 to 0.043)	−0.007 (−0.056 to 0.043)	−0.011 (−0.045 to 0.023)
EN	−0.006 (−0.061 to 0.049)	0.002 (−0.041 to 0.046)	−0.029 (−0.065 to 0.007)	0.031 (−0.019 to 0.080)	0.021 (−0.014 to 0.055)

Abbreviations: DNAmAA, DNA methylation age acceleration; EA, emotional aggression; EN, emotional neglect; PA, physical assault; PN, physical neglect.

^a^ Model was adjusted for age, assay type, cell proportions, internalizing and externalizing problems, sex, city, race and ethnicity, educational level, maternal age, maternal marital status, maternal prenatal smoking, and poverty-to-income ratio (n = 1971).

Analysis of PN and EN found that age 5 years EN was positively associated with PhenoAge age acceleration (β = 0.051; 95% CI, 0.006-0.097), even after adjusting for multiple comparisons, and PN at age 3 years was associated with DunedinPACE age acceleration (β = −0.032; 95% CI, −0.064 to −0.0002), only after accounting for additional ACEs at age 3 years. When examining models with limited controls (eTable 3 in Supplement 1), it was clear that the association for neglect was ameliorated by controls (eg, poverty level).

In the age 3 and 5 years cumulative model, PA was positively associated with PhenoAge age acceleration (β = 0.063; 95% CI, 0.003-0.123), even after adjusting for multiple comparisons. In addition, cumulative EA at ages 3 and 5 years was negatively associated with PhenoAge age acceleration (β = −0.104; 95% CI, −0.165 to −0.043), even after adjusting for multiple comparisons. After additional adjustment for cumulative experience of other ACEs at ages 3 and 5 years (eTable 2 in Supplement 1), cumulative PA at ages 3 and 5 years was associated with PhenoAge age acceleration (β = 0.062; 95% CI, 0.002-0.122). Cumulative EA at ages 3 and 5 years was associated with PhenoAge age acceleration (β = −0.106; 95% CI, −0.167 to −0.044). Results of sensitivity analyses (eTable 4 in Supplement 1) indicated that accumulation of all types of maltreatment between ages 3 and 5 years was not associated with DNAmAA, pointing to the importance of modeling maltreatment accumulation by subtype to avoid masking potential differential associations.

### Child Maltreatment and Epigenetic Aging at Age 15 Years

Including the analyses of age 15 years DNAmAA affords a test of the lasting outcomes of child maltreatment (Table 3). Because the age 9 and 15 years methylation assays were run together, there was no batch effect over time. Results for the age 3 and 5 years associations parallel the results for age 9 years. Physical abuse at age 3 years was positively associated with PhenoAge age acceleration at age 15 years (β = 0.064; 95% CI, 0.009-0.119). In addition, EA at age 3 years was negatively associated with PhenoAge age acceleration at age 15 years (β = −0.085; 95% CI, −0.142 to −0.029), as was EA at age 3 to 5 years (β = −0.065; 95% CI, −0.127 to −0.002). Emotional neglect at age 5 years was positively associated with PhenoAge age acceleration at age 15 years (β = 0.050; 95% CI, 0.003-0.096). After additional adjustment for other ACEs at age 3 years (eTable 5 in Supplement 1), PA at age 3 years was associated with PhenoAge age acceleration at age 15 years (β = 0.065; 95% CI, 0.010-0.120). Emotional abuse at age 3 years was associated with PhenoAge age acceleration at age 15 years (β = −0.082; 95% CI, −0.139 to −0.026), as was EA at ages 3 to 5 years (β = −0.065; 95% CI, −0.128 to −0.002). In the age 5 years model, EN was positively associated with PhenoAge age acceleration at age 15 years (β = 0.050; 95% CI, 0.003-0.096). When accounting for age 9 years accelerated age (eTable 6 in Supplement 1), however, results were attenuated (ie, effect sizes reduced on average by 70%-80%).

**Table 3.** Associations Between Child Maltreatment Exposure and DNA Methylation Age Acceleration Measures at Year 15 (Model 1)^a^

Maltreatment exposure	Year 15 DNAmAA, β (95% CI)				
	Horvath	PedBE	GrimAge	PhenoAge	DunedinPACE
**Year 3**					
PA	0.041 (−0.019 to 0.102)	0.010 (−0.022 to 0.043)	0.017 (−0.020 to 0.054)	0.064 (0.009 to 0.119)	0.017 (−0.019 to 0.053)
EA	−0.049 (−0.111 to 0.013)	0.019 (−0.014 to 0.052)	−0.009 (−0.047 to 0.029)	−0.085 (−0.142 to −0.029)	−0.006 (−0.043 to 0.031)
PN	−0.022 (−0.074 to 0.030)	0.012 (−0.016 to 0.040)	−0.007 (−0.039 to 0.025)	−0.023 (−0.071 to 0.024)	−0.026 (−0.058 to 0.005)
EN	0.013 (−0.039 to 0.065)	−0.009 (−0.037 to 0.019)	−0.008 (−0.040 to 0.024)	0.001 (−0.047 to 0.049)	0.008 (−0.024 to 0.039)
**Year 5**					
PA	0.037 (−0.022 to 0.096)	0.008 (−0.023 to 0.040)	0.008 (−0.027 to 0.044)	−0.030 (−0.082 to 0.023)	0.012 (−0.023 to 0.046)
EA	−0.011 (−0.073 to 0.051)	−0.001 (−0.033 to 0.032)	0.009 (−0.046 to 0.028)	−0.003 (−0.059 to 0.052)	−0.005 (−0.041 to 0.032)
PN	−0.014 (−0.066 to 0.038)	−0.006 (−0.033 to 0.022)	0.011 (−0.020 to 0.042)	0.017 (−0.030 to 0.063)	0.010 (−0.021 to 0.040)
EN	−0.009 (−0.061 to 0.043)	0.013 (−0.015 to 0.040)	−0.013 (−0.044 to 0.018)	0.050 (0.003 to 0.096)	0.007 (−0.024 to 0.037)
**Years 3 and 5**					
PA	0.057 (−0.011 to 0.125)	0.006 (−0.030 to 0.042)	0.013 (−0.028 to 0.054)	0.018 (−0.043 to 0.080)	0.015 (−0.025 to 0.055)
EA	−0.048 (−0.118 to 0.021)	0.014 (−0.023 to 0.051)	−0.010 (−0.052 to 0.032)	−0.065 (−0.127 to −0.002)	−0.004 (−0.045 to 0.037)
PN	−0.031 (−0.087 to 0.025)	0.016 (−0.013 to 0.046)	0.006 (−0.028 to 0.039)	0.003 (−0.047 to 0.053)	0.002 (−0.031 to 0.035)
EN	0.002 (−0.055 to 0.058)	0.003 (−0.026 to 0.033)	−0.020 (−0.054 to 0.014)	0.046 (−0.005 to 0.096)	0.019 (−0.014 to 0.052)

Abbreviations: DNAmAA, DNA methylation age acceleration; EA, emotional aggression; EN, emotional neglect; PA, physical assault; PN, physical neglect.

^a^ Model was adjusted for age, assay type, cell proportions, internalizing and externalizing problems, sex, city, race and ethnicity, educational level, maternal age, maternal marital status, maternal prenatal smoking, and poverty-to-income ratio (n = 1906).

## Discussion

This study elucidates the prospective association between child maltreatment subtypes and DNAmAA in a large cohort representative of births in US cities. First, our results provide evidence that maltreatment is associated with DNAmAA, with alterations evaluated in aging clocks. We consistently found that exposure to PA and EA by the parent was associated with PhenoAge age acceleration, even when accounting for other ACEs. This result contrasts with previous research that identified GrimAge, but not PhenoAge, as sensitive to maltreatment exposure among adult samples,^36,41^ suggesting the importance of the timing of DNAm sampling. PhenoAge may capture precursors to early aging in our childhood sample, while GrimAge may reflect premature aging in adult mortality risks. Overall, these findings emphasize that experiencing abuse during childhood is centrally implicated in second-generation clocks estimating epigenetic aging, potentially tied to major health risks.^29,30,31^

Second, our findings suggest that maltreatment subtypes are differentially associated with patterns of epigenetic age acceleration and deceleration. While physical assault had positive associations with DNAmAA, emotional aggression had negative associations with DNAmAA. This suggests that the unique aspects of emotional abuse that do not overlap with physical abuse may be associated with slower epigenetic aging. It may be that reduced epigenetic aging is capturing the activation of factors that enhance systemic protection in a short-term coping response to psychological stress, which with continued activation may also impart health risks that become evident later in development. Because of the high correlation with PA, it is particularly difficult to isolate the effect. It will be important to replicate these results in other cohort studies, examine associations with emotional abuse and DNAmAA over a longer time, and determine whether there are alternative or delayed biological costs.

Regarding experiences of neglect, after accounting for key covariates and multiple comparisons, there was little evidence linking PN or EN with DNAmAA. This may be because measures of neglect are highly correlated with poverty; substantial limitations on resources can make it unclear whether the parents are neglectful or the context of poverty does not afford parents sufficient resources. This finding complicates previous findings that have indicated an association between deprivation-related adversities and delayed biological aging metrics,^42^ suggesting that different types of deprivation (ie, PN vs EN) might have distinct associations with biological aging.

The differential patterns of DNAmAA for maltreatment subtypes observed in the present study suggest that composite scores of adversity may dilute our ability to detect the outcomes of maltreatment associated with epigenetic aging in childhood and underline the importance of investigating specific maltreatment subtype association with epigenetic age. In line with this view, our study expands on previous work with mixed results concerning the association between neglect and epigenetic aging. Future studies that examine the discrete impacts of different forms of neglect using improved measures are encouraged.^54,55^

Third, we sought to examine whether our results aligned with a developmental sensitivity model, an accumulation model, or both. Our findings when assessing maltreatment exposure independently at ages 3 and 5 years compared with that accumulated across ages 3 to 5 years point to some notable differences. Developmental sensitivity models showed that maltreatment at age 3 years (ie, PA, EA, and PN) was associated with DNAmAA at age 9 years; however, only 1 form of maltreatment at age 5 years (ie, EN) was associated with DNAmAA at age 9 years. This is consistent with research indicating that ACEs by age 3 years were most saliently associated with altered DNAm at age 7 years.^44^ This finding is notable and concerning, given that maltreatment rates peak in the first 3 years of life.^56^

The accumulation of physical and emotional maltreatment between ages 3 and 5 years was also associated with epigenetic aging at age 9 years. While previous studies have often focused on the accumulation of ACEs on DNAm at specific sites,^9,44^ our results extend this by showing an additive impact of maltreatment events experienced in early childhood associated with epigenetic aging, even after accounting for other ACEs. As theorized life course models of exposure patterns are not mutually exclusive (one may have both sensitive periods and accumulating exposures), our findings support moving beyond binary notions of exposure to child maltreatment toward more specific analyses of how timing and accumulation of exposure to child maltreatment at different stages across development may account for variations in associations with DNAmAA.

Fourth, although we found the outcomes of child maltreatment at age 3 years remain even until age 15 years, the association appeared to be considerably mediated by age 9 years DNAmAA. This suggests that while the biological embedding of maltreatment is significant, it may not necessarily continue to have a biological impact, suggesting that the biological embedding of stress may be able to be arrested.

### Limitations

This study has limitations. First, while the present urban sample has greater representation of Black and low-income families, it would be important to examine whether these findings are generalizable to more rural settings in which families face unique risk and protective factors that may influence the risk for and consequences of maltreatment.^57^ Second, relying on parent or caregiver reports of maltreatment may result in underestimation. Future work should examine whether important differences emerge when analyzing self-reported and substantiated cases of maltreatment. Third, as with all longitudinal studies, attrition occurred in this sample. However, our analytic sample did not meaningfully differ from that of baseline participant characteristics. Fourth, considering the small effect sizes observed, future research should explore moderating factors that could amplify or mitigate the association between child maltreatment and epigenetic aging.^58,59^

## Conclusions

This prospective study of a national birth cohort found that changes in DNAm (both acceleration and deceleration) appeared to be sensitive to the type and timing of maltreatment. Physical and emotional abuse, as well as PN at age 3 years and EN at age 5 years, were associated with epigenetic signatures of aging. The findings suggest that child maltreatment appears to have a lasting influence through more proximate biological embedding of stress.

## Notes

Supplement 1. **eMethods.** Sample Collection, Storage, and Processing for FFCW Saliva Samples **eTable 1.** Demographic Characteristics of Overall FFCWS Sample and DNA Assay Subsample **eTable 2.** Associations Between Child Maltreatment Exposure and DNA Methylation Age Acceleration Measures at Year 9 (Model 2) **eTable 3.** Associations Between Child Maltreatment Exposure and DNA Methylation Age Acceleration Measures at Year 9 (Model 0) **eTable 4.** Sensitivity Test of Child Maltreatment Exposure and DNA Methylation Age Acceleration Measures at Year 9 **eTable 5.** Associations Between Child Maltreatment Exposure and DNA Methylation Age Acceleration Measures at Year 15 (Model 2) **eTable 6.** Associations Between Child Maltreatment Exposure and DNA Methylation Age Acceleration Measures at Year 15 After Additional Adjustment DNA Methylation Age Acceleration at Year 9 (Model 3)

Supplement 2.
Data Sharing Statement
