# Supplementary material for: Childhood Maltreatment and Longitudinal Epigenetic Aging: NIMHD Social Epigenomics Program
Source: JAMA Netw Open. 2024 Jul 29;7(7):e2421877. doi: 10.1001/jamanetworkopen.2024.21877 (PMC11287393; doi:10.1001/jamanetworkopen.2024.21877)
Supplement: Supplement 1. — eMethods. Sample Collection, Storage, and Processing for FFCW Saliva Samples eTable 1. Demographic Characteristics of Overall FFCWS Sample and DNA Assay Subsample eTable 2. Associations Between Child Maltreatment Exposure and DNA Methylation Age Acceleration Measures at Year 9 (Model 2) eTable 3. Associations Between Child Maltreatment Exposure and DNA Methylation Age Acceleration Measures at Year 9 (Model 0) eTable 4. Sensitivity Test of Child Maltreatment Exposure and DNA Methylation Age Acceleration Measures at Year 9 eTable 5. Associations Between Child Maltreatment Exposure and DNA Methylation Age Acceleration Measures at Year 15 (Model 2) eTable 6. Associations Between Child Maltreatment Exposure and DNA Methylation Age Acceleration Measures at Year 15 After Additional Adjustment DNA Methylation Age Acceleration at Year 9 (Model 3) [file jamanetwopen-e2421877-s001.pdf]

Supplementary Online Content

Chang OD, Meier HCS, Maguire-Jack K, Davis-Kean P, Mitchell C. Childhood maltreatment and longitudinal epigenetic aging: NIMHD Social Epigenomics Program. *JAMA Netw Open*. 2024;7(7):e2421877. doi:10.1001/jamanetworkopen.2024.21877

eMethods. Sample Collection, Storage, and Processing for FFCW Saliva Samples

eTable 1. Demographic Characteristics of Overall FFCWS Sample and DNA Assay Subsample

eTable 2. Associations Between Child Maltreatment Exposure and DNA Methylation Age Acceleration Measures at Year 9 (Model 2)

eTable 3. Associations Between Child Maltreatment Exposure and DNA Methylation Age Acceleration Measures at Year 9 (Model 0)

eTable 4. Sensitivity Test of Child Maltreatment Exposure and DNA Methylation Age Acceleration Measures at Year 9

eTable 5. Associations Between Child Maltreatment Exposure and DNA Methylation Age Acceleration Measures at Year 15 (Model 2)

eTable 6. Associations Between Child Maltreatment Exposure and DNA Methylation Age Acceleration Measures at Year 15 After Additional Adjustment DNA Methylation Age Acceleration at Year 9 (Model 3)

This supplementary material has been provided by the authors to give readers additional information about their work.

© 2024 Chang OD et al. *JAMA Network Open.*

eMethods. Sample Collection, Storage, and Processing for FFCW Saliva Samples

As part of the Year 9 follow-up wave, we attempted to collect saliva samples for genetic analysis from all focal children and biological mothers completing the in-home visit activities. In cases where a biological father or non-parental figure was the primary caregiver, or the biological mother was not present for the in-home visit, a saliva sample was collected from only the child. Families completing the home visit activities received a $65 payment to the primary caregiver and a $30 payment to the child. No additional remuneration was provided specifically for the contribution of saliva samples. Ultimately, 3,403 in-home visits were conducted; 2,884 unique child samples and 2,670 unique mother samples were collected.

The FFCWS survey subcontractor, Westat Inc., arranged sample collection. Westat interviewers used the Oragene® DNA Self-Collection Kit to collect saliva samples from focal children and biological mothers during the Home Visit. The Self-Collection Kit is a repository for the collection, preservation, and transportation of saliva. The respondents were instructed to spit into the container until the liquid portion reached a line on the interior of the container (the ideal volume of saliva to be collected was 2 ml). The container was then capped. In the process of screwing the cap onto the container, a liquid preservative was released. The container was then put into a small plastic biohazard bag that contained absorbent material if the container were to leak. The plastic bag was then put into a mailer. In cases where the child had developmental or physical limitations prohibiting the interviewer from collecting the full sample by having the child spit into the collection kit, the child accessory kit was used. The child accessory kit contained a set of five saliva sponges used with the Oragene® self-collection kits. The saliva sponges were inserted into the child's mouth and moved around the upper and lower cheek pouches on both sides of the mouth to collect saliva. The sponges were stored inside the containers and then sealed as described above. Respondents were instructed to rinse their mouth out 5 minutes prior to the saliva sample collection. They were also provided with a packet of sugar and instructed to use ¼ tsp. if they were having difficulty stimulating saliva. After completing a Home Visit, interviewers mailed the specimen containers (placed in the bubble wrap mailers) back to Westat.

During the Year 15 follow-up wave, saliva was collected from the focal children (now teenagers) using Oragene DNA Self-Collection Kits (OGR-500) as described for the year 9 follow-up with the following modifications. For those who did not complete a home visit, saliva collection kits were sent to participants via mail and after collection participants returned the kits to Westat via FedEx. Participants were discouraged from eating or drinking anything within 30 minutes prior to sample collection. Upon completion of the saliva collection, all participants received $20.

As Westat received specimen containers from the field, they were inspected to make sure that samples did not contain personal identifiers and placed in a shipment box with other received collection kits. Until they were mailed, these boxes were secured in the locked field room and maintained at room temperature. On an approximately monthly basis during the field period, Westat shipped boxes of specimen containers at room temperature to the laboratory of Dr. Daniel Notterman, Co-Principal Investigator of the FFCWS, in the Department of Molecular Biology at Princeton University. A transmittal form containing the IDs of the enclosed containers was

© 2024 Chang OD et al. *JAMA Network Open.*

emailed to lab staff. The lab confirmed receipt of the boxes with Westat. Saliva collection kits were shipped monthly from October 2007 through May 2010 and from April 2014 through March 2017, respectively, for the 9- and 15- year follow-ups to the Notterman laboratory at Princeton by FedEx from Westat. Upon receipt of the shipments, lab technicians used a barcode reader to inventory the individual samples. These data were imported into a Microsoft Access database where a full inventory of receipted samples is kept.

Extraction was completed 1 to 2 weeks after receipt of samples from Westat. DNA was extracted from the entire sample using the Oragene® prepIT•L2P Laboratory Protocol for Manual Purification of DNA (DNA Genotek). Briefly, when samples were ready to be processed, they were incubated at 50°C in a water incubator for a minimum of 1 hour. The mixed Oragene®-DNA/saliva sample was transferred to a 15 ml centrifuge tube. A 1/25 ul volume portion of Oragene®- prepIT•L2P solution was added to the microcentrifuge tube and mixed by vortexing for a few seconds. The sample was incubated on ice for 10 minutes, then centrifuged at room temperature for 10 minutes at 3,500 rpm. The clear supernatant was carefully transferred with a pipet into a fresh centrifuge tube, avoiding the precipitate at the bottom of the tube. A volume of room temperature 100% ethanol equal to the volume of the supernatant was added to the supernatant and gently mixed by inversion 10 times. The sample was allowed to stand for 10 minutes at room air to allow the DNA to fully precipitate. The tube was then centrifuged for 10 minutes at room temperature at 3,500 rpm. The supernatant was decanted and discarded, taking care to avoid disturbing the DNA pellet. An ethanol wash consisting of 1 ml of 70% ethanol was added to the tube without disturbing the pellet. After standing at room temperature for 1 minute, the tube was gently swirled to completely remove the ethanol, taking care not to disturb the pellet. The pellet was air dried after which the DNA was rehydrated by adding 0.5 to 1.0 ml of TE solution (10 mM Tris-HCl, 1 mM EDTA, pH 7.5), vortexing the sample for 30 seconds, incubating it at room temperature, and transferring the rehydrated DNA to 3 x 1.7 ml microcentrifuge tubes for storage (2 tubes were stored at -80°C and one in the lab refrigerator at 4°C). DNA concentration was determined using the Quant-iT PicoGreen dsDNA Assay Kit (ThermoFisher).

Approximately 500 ng of genomic DNA (quantified using the Quant-iT Picogreen ds DNA Assay Kit as described above) was subjected to bisulfite conversion using the EZ-96 DNA Methylation Kit (Zymo Research) and analyzed with the Illumina Infinium Human Methylation450K (450K) or Illumina Infinium MethylationEPIC (EPIC) array according to the manufacturer's protocol. Bisulfite conversion and array processing was performed by the Pennsylvania State College of Medicine Genome Sciences Core facility. Age 9 and 15 samples were run at the same time to minimize technical variation. Otherwise, samples were randomized. The red and green image pairs were read into R. QC of the methylation data was initially performed with EWAStools. Probes were removed if the detection value was greater than 0.01 or 0.05 for the 450K or EPIC arrays, respectively. Probes were also removed if the number of methylated or unmethylated bead count was fewer than four. Probes were also removed if they were identified by the ENmix function QCinfo using the default parameters. Samples were removed if they had outlier methylation or bisulfite conversion values, as identified by the ENmix QC function or if the sex predicted from the methylation data differed

from the recorded sex. If the sequential samples from the same individual exhibited genetic discordance between visits the sample was flagged. The ENmix preprocessENmix and rcp functions were used to normalize dye bias, apply background correction and adjust for probe-type bias.

© 2024 Chang OD et al. *JAMA Network Open.*

eTable 1. Demographic Characteristics of Overall FFCWS Sample and DNA Assay Subsample

Variables	Overall FFCWS (*n* = 4,898)	Age 9 DNA Assay (*n* = 1,971)	Age 9 and 15 DNA Assay (*n* = 1,906)
Child sex			
Male	2,556 (52.2)	992 (50.3)	959 (50.3)
Female	2,341 (47.8)	979 (49.7)	947 (49.7)
Race/ethnicity			
Black, non-Hispanic	2,166 (44.2)	889 (45.1)	856 (44.9)
Hispanic	1,124 (23.0)	425 (21.6)	408 (21.4)
Multiracial[a]	728 (14.9)	281 (14.3)	276 (14.5)
White, non-Hispanic	784 (16.0)	344 (17.5)	334 (17.5)
Other	96 (2.0)	32 (1.6)	32 (1.7)
Mother's Education			
Less than high school	1,699 (34.7)	616 (31.3)	599 (31.5)
High school degree or GED	1,480 (30.2)	607 (30.9)	576 (30.3)
Some college or technical school	1,189 (24.3)	520 (26.4)	507 (26.7)
College degree or higher	524 (10.7)	224 (11.4)	220 (11.6)
Poverty-to-income ratio	2.22 (2.41)	2.34 (2.49)	2.36 (2.50)

[a]Multiracial includes children whose parents reported more than one race/ethnicity.

© 2024 Chang OD et al. *JAMA Network Open.*

eTable 2. Associations Between Child Maltreatment Exposure and DNA Methylation Age Acceleration Measures at Year 9 (Model 2[a])

Maltreatment Exposure	Year 9 DNAmAA				
	Horvath	PedBE	GrimAge	PhenoAge	DunedinPACE
	β (95% CI)	β (95% CI)	β (95% CI)	β (95% CI)	β (95% CI)
Year 3					
PA	.029 (-.030, .088)	.001 (-.048, .049)	.028 (-.011, .067)	.074 (.020, .128)	.015 (-.022, .052)
EA	-.042 (-.103, .018)	.054 (.004, .103)	-.012 (-.053, .028)	-.105 (-.160, -.049)	-.002 (-.041, .036)
PN	-.053 (-.104, -.002)	.009 (-.033, .050)	-.004 (-.038, .029)	.001 (-.045, .048)	-.032 (-.064, <-.001)
EN	-.002 (-.054, .049)	-.001 (-.043, .042)	-.023 (-.057, .011)	-.010 (-.057, .037)	.006 (-.026, .039)
Year 5					
PA	.036 (-.021, .094)	-.001 (-.047, .045)	.020 (-.017, .057)	.017 (-.036, .069)	.002 (-.034, .037)
EA	-.035 (-.096, .025)	.030 (-.018, .078)	-.013 (-.051, .026)	-.035 (-.090, .019)	-.010 (-.048, .027)
PN	-.022 (-.073, .028)	-.012 (-.052, .028)	.020 (-.012, .053)	.005 (-.040, .051)	-.015 (-.046, .016)
EN	-.004 (-.054, .047)	.004 (-.036, .045)	-.014 (-.047, .018)	.051 (.005, .010)	.021 (-.010, .052)
Years 3 and 5					
PA	.051 (-.015, .116)	<.001 (-.052, .053)	.039 (-.004, .0082)	.062 (.002, .122)	.011 (-.031, .052)
EA	-.060 (-.128, .008)	.059 (.004, .113)	-.017 (-.062, .027)	-.106 (-.167, -.044)	-.006 (-.049, .036)
PN	-.024 (-.079, .031)	.016 (-.027, .060)	.011 (-.025, .046)	-.008 (-.057, .042)	-.010 (-.044, .025)
EN	-.004 (-.059, .051)	.005 (-.039, .049)	-.026 (-.062, .009)	.030 (-.020, .080)	.022 (-.013, .056)

Abbreviations: PA = Physical Assault; EA = Emotional Aggression; PN = Physical Neglect; EN = Emotional Neglect; CI = Confidence Interval. [a]Model is adjusted for age, assay type, cell proportions, internalizing/externalizing problems, sex, city, race/ethnicity, education, maternal age, maternal marital status, maternal prenatal smoking, poverty-to-income ratio, and other ACEs. *n* = 1,971.

© 2024 Chang OD et al. *JAMA Network Open.*

eTable 3. Associations Between Child Maltreatment Exposure and DNA Methylation Age Acceleration Measures at Year 9 (Model 0[a])

Maltreatment Exposure	Year 9 DNAmAA				
	Horvath	PedBE	GrimAge	PhenoAge	DunedinPACE
	β (95% CI)	β (95% CI)	β (95% CI)	β (95% CI)	β (95% CI)
Year 3					
PA	.031 (-.026, .089)	.029 (-.019, .077)	.060 (.021, .010)	.076 (.022, .130)	.029 (-.009, .066)
EA	-.046 (-.103, .012)	.057 (.009, .104)	-.002 (-.042, .037)	-.111 (-.165, -.058)	-.015 (-.052, .022)
PN	-.052 (-.103, -.002)	.012 (-.031, .054)	-.006 (-.040, .029)	.014 (-.033, .062)	-.026 (-.059, .006)
EN	-.006 (-.057, .044)	-.005 (-.048, .037)	-.017 (-.052, .017)	-.013 (-.061, .035)	.006 (-.026, .039)
Year 5					
PA	.033 (-.023, .089)	.021 (-.025, .066)	.045 (.007, .082)	.016 (-.035, .068)	.016 (-.020, .051)
EA	-.043 (-.010, .013)	.025 (-.021, .071)	.005 (-.033, .042)	-.067 (-.119, -.015)	-.010 (-.046, .025)
PN	-.020 (-.071, .030)	-.003 (-.044, .038)	.023 (-.010, .057)	.013 (-.034, .060)	-.010 (-.042, .022)
EN	-.004 (-.054, .047)	-.005 (-.046, .036)	-.009 (-.043, .024)	.055 (.008, .101)	.029 (-.003, .061)
Years 3 and 5					
PA	.048 (-.015, .112)	.025 (-.026, .077)	.075 (.032, .118)	.064 (.005, .123)	.030 (-.011, .071)
EA	-.061 (-.124, .002)	.054 (.003, .106)	-.005 (-.047, .038)	-.123 (-.182, -.065)	-.015 (-.055, .026)
PN	-.026 (-.080, .028)	.022 (-.022, .065)	.011 (-.025, .047)	.005 (-.045, .055)	.002 (-.033, .037)
EN	-.002 (-.056, .052)	-.011 (-.054, .033)	-.020 (-.056, .016)	.034 (-.016, .084)	.027 (-.008, .061)

Abbreviations: PA = Physical Assault; EA = Emotional Aggression; PN = Physical Neglect; EN = Emotional Neglect; CI = Confidence Interval. [a]Model is adjusted for age, assay type, and cell proportions. n = 1,971.

© 2024 Chang OD et al. *JAMA Network Open.*

eTable 4. Sensitivity Test of Child Maltreatment Exposure and DNA Methylation Age Acceleration Measures at Year 9[a]

Maltreatment Exposure	Year 9 DNAmAA				
	Horvath	PedBE	GrimAge	PhenoAge	DunedinPACE
	β (95% CI)	β (95% CI)	β (95% CI)	β (95% CI)	β (95% CI)
Years 3 and 5					
AM	-.022 (-.083, .039)	.049 (<-.001, .097)	.008 (-.032, .048)	-.041 (-.097, .014)	.008 (-.031, .046)

Abbreviations: AM = All maltreatment types. CI = Confidence Interval. [a]Model is adjusted for age, assay type, cell proportions, internalizing/externalizing problems, sex, city, race/ethnicity, education, maternal age, maternal marital status, maternal prenatal smoking, and poverty-to-income ratio. *n* = 1,971.

© 2024 Chang OD et al. *JAMA Network Open.*

eTable 5. Associations Between Child Maltreatment Exposure and DNA Methylation Age Acceleration Measures at Year 15 (Model 2[a])

Maltreatment Exposure	Year 15 DNAmAA				
	Horvath	PedBE	GrimAge	PhenoAge	DunedinPACE
	β (95% CI)	β (95% CI)	β (95% CI)	β (95% CI)	β (95% CI)
Year 3					
PA	.043 (-.017, .103)	.011 (-.021, .043)	.017 (-.020, .054)	.065 (.010, .120)	.018 (-.019, .054)
EA	-.045 (-.107, .017)	.020 (-.013, .054)	-.008 (-.047, .030)	-.082 (-.139, -.026)	-.004 (-.041, .033)
PN	-.022 (-.074, .030)	.012 (-.016, .039)	-.007 (-.039, .025)	-.024 (-.071, .024)	-.027 (-.058, .004)
EN	.017 (-.035, .070)	-.008 (-.036, .020)	-.008 (-.040, .025)	.004 (-.044, .052)	.009 (-.022, .041)
Year 5					
PA	.037 (-.022, .096)	.009 (-.023, .040)	.009 (-.026, .044)	-.030 (-.083, .023)	.012 (-.023, .047)
EA	-.011 (-.073, .051)	<-.001 (-.033, .032)	-.008 (-.045, .029)	-.004 (-.059, .051)	-.004 (-.041, .032)
PN	-.014 (-.066, .038)	-.006 (-.034, .022)	.011 (-.021, .042)	.017 (-.030, .063)	.010 (-.021, .040)
EN	-.009 (-.061, .043)	.013 (-.015, .041)	-.013 (-.044, .018)	.050 (.003, .096)	.007 (-.024, .037)
Years 3 and 5					
PA	.059 (-.008, .127)	.007 (-.029, .043)	.014 (-.027, .055)	.018 (-.043, .080)	.017 (-.024, .056)
EA	-.044 (-.114, .026)	.015 (-.022, .052)	-.008 (-.051, .034)	-.065 (-.128, -.002)	-.001 (-.043, .040)
PN	-.027 (-.084, .029)	.017 (-.013, .047)	.007 (-.026, .041)	.003 (-.047, .054)	.004 (-.029, .037)
EN	.005 (-.051, .062)	.004 (-.026, .034)	-.018 (-.052, .015)	.046 (-.005, .097)	.021 (-.012, .054)

Abbreviations: PA = Physical Assault; EA = Emotional Aggression; PN = Physical Neglect; EN = Emotional Neglect; CI = Confidence Interval. [a]Model is adjusted for age, assay type, cell proportions, internalizing/externalizing problems, sex, city, race/ethnicity, education, maternal age, maternal marital status, maternal prenatal smoking, poverty-to-income ratio, and other ACEs. *n* = 1,906.

© 2024 Chang OD et al. *JAMA Network Open.*

eTable 6. Associations Between Child Maltreatment Exposure and DNA Methylation Age Acceleration Measures at Year 15 After Additional Adjustment DNA Methylation Age Acceleration at Year 9 (Model 3[a])

Maltreatment Exposure	Year 15 DNAmAA				
	Horvath	PedBE	GrimAge	PhenoAge	DunedinPACE
	β (95% CI)	β (95% CI)	β (95% CI)	β (95% CI)	β (95% CI)
Year 3					
PA	.003 (-.023, .029)	.011 (-.014, .037)	-.005 (-.031, .021)	.016 (-.027, .060)	.009 (-.021, .040)
EA	<-.001 (-.027, .027)	-.002 (-.027, .024)	<.001 (-.027, .027)	-.018 (-.063, .027)	-.001 (-.032, .031)
PN	.032 (.009, .054)	.007 (-.015, .028)	-.004 (-.026, .018)	-.023 (-.060, .015)	-.012 (-.039, .014)
EN	.005 (-.017, .028)	-.006 (-.028, .016)	.009 (-.013, .032)	.006 (-.032, .044)	.008 (-.019, .035)
Year 5					
PA	.001 (-.025, .027)	.011 (-.013, .036)	-.008 (-.033, .017)	-.042 (-.083, <-.001)	.009 (-.021, .039)
EA	.018 (-.010, .045)	-.014 (-.040, .011)	-.003 (-.029, .023)	.015 (-.028, .058)	.002 (-.030, .033)
PN	-.003 (-.026, .020)	.001 (-.021, .022)	-.005 (-.026, .017)	.010 (-.026, .047)	.019 (-.007, .045)
EN	.001 (-.022, .024)	.010 (-.011, .032)	-.001 (-.023, .021)	.018 (-.018, .055)	-.004 (-.030, .022)
Years 3 and 5					
PA	-.001 (-.030, .029)	.008 (-.020, .036)	-.018 (-.047, .011)	-.022 (-.070, .025)	.010 (-.024, .044)
EA	.013 (-.018, .043)	-.011 (-.040, .018)	.002 (-.027, .032)	-.003 (-.053, .046)	.002 (-.033, .037)
PN	-.005 (-.029, .020)	.009 (-.014, .033)	.001 (-.023, .025)	.007 (-.032, .047)	.009 (-.019, .038)
EN	.006 (-.019, .030)	.001 (-.022, .025)	.002 (-.022, .026)	.026 (-.014, .066)	.012 (-.017, .040)

Abbreviations: PA = Physical Assault; EA = Emotional Aggression; PN = Physical Neglect; EN = Emotional Neglect; CI = Confidence Interval. [a]Model is adjusted for age, assay type, cell proportions, internalizing/externalizing problems, sex, city, race/ethnicity, education, maternal age, maternal marital status, maternal prenatal smoking, poverty-to-income ratio, other ACEs, and DNA methylation age acceleration at Year 9. n = 1,906.

© 2024 Chang OD et al. *JAMA Network Open.*
