# Supplementary material for: Childhood Maltreatment and Longitudinal Epigenetic Aging: NIMHD Social Epigenomics Program
Source: JAMA Netw Open. 2024 Jul 29;7(7):e2421877. doi: 10.1001/jamanetworkopen.2024.21877 (PMC11287393; doi:10.1001/jamanetworkopen.2024.21877)
Supplement: Supplement 2. — Data Sharing Statement [file jamanetwopen-e2421877-s002.pdf]

Data Sharing Statement

Chang. Childhood Maltreatment and Longitudinal Epigenetic Aging. *JAMA Netw Open*.
Published July 29, 2024. doi:10.1001/jamanetworkopen.2024.21877

Data
Data available: Yes
Data types: Deidentified participant data
How to access data: Data from the Future Families and Child Wellbeing Study is publicly available at: https://ffcws.princeton.edu/
When available: With publication

Supporting Documents
Document types: None

Additional Information
Who can access the data: Researchers who request the data
Types of analyses: For any research purpose
Mechanisms of data availability: with a signed data access agreement
